# Determinants of Microbial Resistance to Far-UVC 222 nm in Healthcare Pathogens: A Narrative Review

**DOI:** 10.3390/life16050842

**Published:** 2026-05-19

**Authors:** Septika Prismasari, Jung Yun Kang

**Affiliations:** 1 Department of Dental Hygiene, College of Health Sciences, Yonsei University, Wonju 26493, Republic of Korea; septika.p@yonsei.ac.kr; 2 Department of Preventive and Community Dentistry, Faculty of Dentistry, Universitas Gadjah Mada, Yogyakarta 55281, Indonesia

**Keywords:** bacterial resistance, biofilm, disinfection, far-UVC 222 nm, healthcare-associated infections, pathogen, Ultraviolet C

## Abstract

Far-UVC 222 nm is a promising adjunctive disinfection technology for occupied healthcare environments, though antimicrobial efficacy varies significantly across pathogen types due to fundamental differences in microbial biology. This review synthesizes evidence on microbiological determinants of far-UVC resistance, examining cell envelope structure, biofilm formation, DNA repair capacity, and antioxidant defenses. A clear resistance hierarchy emerges. Enveloped viruses lacking enzymatic repair systems are highly vulnerable, requiring fluences below 3 mJ/cm^2^. Gram-negative bacteria are readily inactivated through membrane disruption and reactive oxygen species accumulation. Gram-positive bacteria demonstrate higher resistance via thick peptidoglycan barriers, DNA repair mechanisms, and redundant antioxidant systems. Biofilm-embedded cells show 10–1000-fold increased tolerance due to protective extracellular matrices, stress-response gene upregulation, and microenvironmental heterogeneity. *Clostridioides difficile* spores exhibit extreme resistance through multilaminar protective coats and metabolic dormancy, requiring impractical doses exceeding 1000 mJ/cm^2^. Field studies in real-world polymicrobial biofilm communities demonstrate substantially lower efficacy than laboratory predictions, typically achieving only 55–81% bioburden reductions. Understanding these pathogen-specific resistance mechanisms is essential for the rational deployment of far-UVC as an adjunctive infection prevention intervention in healthcare settings.

## 1. Introduction

Healthcare-associated infections (HAIs) remain a substantial global health concern, with an estimated 136 million cases annually, resulting in severe morbidity, mortality, and economic repercussions [1,2]. Point prevalence surveys indicate that approximately 1 in 31 hospitalized patients acquire at least one healthcare-associated infection. The predominant conditions include pneumonia, surgical site infection, and bloodstream infection [3]. A considerable percentage of these infections are due to multidrug-resistant (MDR) pathogens, such as methicillin-resistant *Staphylococcus aureus* (MRSA), *Pseudomonas aeruginosa*, *Acinetobacter baumannii*, vancomycin-resistant enterococci (VRE), *Candida auris*, and *Clostridioides difficile*, which persist on environmental surfaces and in the air for prolonged periods and frequently reside in biofilms [4,5,6].

Traditional infection control measures, such as contact precautions, negative-pressure isolation, and chemical surface disinfection, are constrained by reliance on human behavior, inadequate coverage of frequently touched surfaces, and variable compliance with protocols [7,8,9]. These primary interventions, including conventional germicidal UVC (254 nm) systems that require room vacancy during operation, chemical disinfection agents such as hypochlorite-based solutions, hydrogen peroxide vapor, and quaternary ammonium compounds, and manual cleaning with mechanical removal of organic soil, remain the established cornerstones of environmental decontamination [7,8,9,10,11]. Environmental studies reveal that a considerable percentage of high-risk items in patient care areas are inadequately sanitized, and the predominant dependence on detergents is entrenched in routine practice [10,11]. These deficiencies persist despite the increasing prevalence of antibiotic resistance, underscoring the need for further complementary disinfection technologies that operate continuously and autonomously from human compliance [12].

Far-ultraviolet C light at 222 nm (far-UVC 222 nm) has emerged as a viable adjunctive disinfection method for occupied spaces. As an adjunctive technology, far-UVC 222 nm is intended to complement rather than replace the primary disinfection modalities described above, providing an additional continuous layer of pathogen reduction in environments where conventional methods alone have proven insufficient [7,12]. Filtered 222 nm radiation is significantly absorbed by the outer non-viable layers of skin and the tear film, enabling sustained operation within existing exposure guidelines while maintaining antimicrobial efficacy against airborne and surface-bound pathogens [13,14,15]. Experimental and field studies have demonstrated that far-UVC 222 nm can effectively inactivate airborne viruses, including human coronaviruses and influenza A virus, along with clinically significant bacteria and fungi on various surfaces [16,17,18,19,20]. However, the recorded fluence needs and log-reduction values demonstrate considerable variability between pathogen categories and environmental conditions.

This variety reflects essential microbial biology. The vulnerability of pathogens to far-UVC 222 nm is determined by the structure of the cell envelope, the ability to form biofilms, DNA repair systems, antioxidant defenses, and the physical environment in which organisms are situated such as airborne aerosol, hydrated surface, dried deposit, or mature biofilm [21,22]. Because the efficacy of far-UVC 222 nm varies substantially with these biological determinants, rational deployment requires an understanding of which pathogens are readily inactivated at practical exposure levels, which require elevated fluences or extended exposure, and which necessitate combined interventions incorporating chemical disinfection, manual cleaning, or conventional UVC alongside far-UVC 222 nm [7,8,20].

This narrative review integrates contemporary research regarding the disinfection efficacy of far-UVC 222 nm against HAIs, highlighting the microbiological factors that influence resistance. The study delineates the microbiological attributes and resistance profiles of principal pathogens, encapsulates mechanistic elucidations of far-UVC 222 nm-induced damage in viruses, bacteria, and fungi, and compiles quantitative efficacy data pertaining to airborne and surface applications.

## 2. Methods

This study was conducted as a narrative review following established methodological guidance for narrative synthesis. Systematic, topic-specific searches were conducted on the Web of Science, Scopus, and Google Scholar to identify peer-reviewed papers assessing the efficacy of far-UVC 222 nm against HAIs published from 1 January 2015 to 31 August 2025. The search technique integrated the phrases “Far UVC,” “Far-UVC,” “Far UV-C,” “Far UVC 222 nm,” “Far-UVC 222 nm,” and “Far 222 nm,” used Boolean operators, phrase searching, and truncation in an iterative manner. Searches were performed from August to September 2025, including solely English-language records accessible online. Additional relevant studies identified during the peer review process were incorporated into the revised manuscript.

Studies qualified for inclusion if they assessed far-UVC 222 nm in healthcare-related settings (in vitro, in situ, or field/room studies), presented quantitative efficacy outcomes (such as percentage reduction, log reduction, or inactivation kinetics), identified organisms to at least the species level when feasible, and explicitly defined the experimental matrix (airborne, surface, or biofilm) and study design. Research concentrating solely on alternative UV wavelengths (excluding 254 nm as a direct comparator), non-pathogenic outcomes like toxicity or lamp physics, or non-healthcare applications such as food or water treatment were omitted, along with review and opinion articles, which served solely as contextual background.

**Figure 1:**
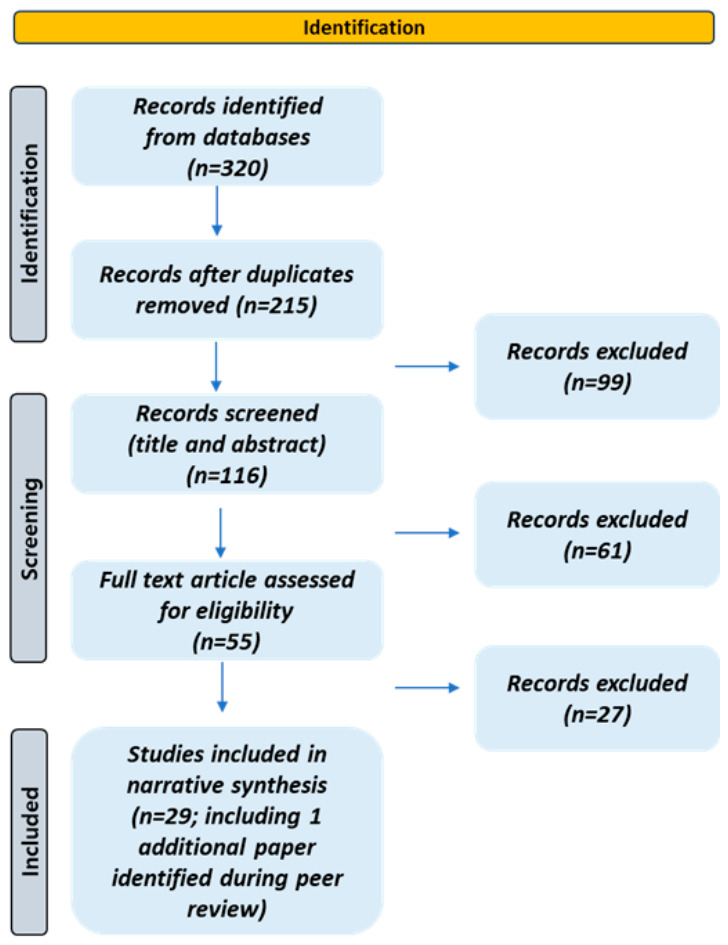
Descriptive flow diagram for study selection.

The initial search yielded approximately 320 records across all three databases. After removing duplicates and screening titles and abstracts, 55 articles underwent full-text evaluation. Of these, 28 studies met the inclusion criteria and were included in the narrative synthesis. One additional study identified during the peer review process was subsequently incorporated, bringing the total to 29 studies included in the final review (Figure 1). The selection process prioritized studies with clearly defined fluence parameters, validated microbiological endpoints, and healthcare-relevant pathogen species or environmental matrices. Because this review was designed with a narrative objective, the evidence was synthesized according to its relevance rather than through systematic data extraction. Consequently, a formal risk-of-bias assessment and quantitative meta-analysis were not performed.

It should be noted that substantial heterogeneity exists among the included studies regarding experimental conditions, including differences in inoculum preparation and titer, surface material and topography, organic load, relative humidity, fluence measurement methodology, and irradiance calibration. These methodological differences complicate direct quantitative comparisons across studies and should be considered when interpreting the synthesized efficacy data presented in the following sections.

## 3. Results

### 3.1. Microbial Characteristics and Healthcare Pathogen Resistance Profiles

Healthcare environments contain various pathogens that demonstrate distinct susceptibilities to far-UVC 222 nm, reflecting inherent differences in cell membrane structure, biofilm development capacity, stress-response systems, and environmental durability. Table 1 shows important healthcare-associated pathogens sorted by the type of microbe, the type of envelope, the ability to form biofilms, the mechanisms for repairing DNA, defenses against free radicals, the ability to survive in different environments, and the predominant healthcare-associated infection syndromes. This configuration underscores that the traits promoting long-term survival on hospital surfaces and providing resistance to antimicrobials similarly influence susceptibility to UV-based disinfection [4,8,21].

At the low-resistance end of the spectrum, enveloped viruses such as influenza A virus and severe acute respiratory syndrome coronavirus 2 (SARS-CoV-2) possess lipid envelopes and lack enzymatic repair systems, making their infectivity highly dependent on intact surface proteins and genomes [17,23]. These viruses display moderate persistence on surfaces, surviving 24 to 48 h for influenza A virus and 4 to 7 days for SARS-CoV-2, and lack the metabolic machinery to repair or counteract photon-induced protein and nucleic acid damage [23,24]. Gram-negative bacteria, including *Escherichia coli* and *Pseudomonas aeruginosa*, are more resistant than viruses; yet remain comparatively vulnerable owing to their lipopolysaccharide outer membranes, which are inherently sensitive to oxidative damage, and relatively thin peptidoglycan layers supported by moderate antioxidant defenses [25,26].

Furthermore, Gram-positive bacteria, such as *Staphylococcus aureus* and *Enterococcus faecium*, demonstrate significantly greater resistance, facilitated by thick peptidoglycan cell walls (20 to 35 nm), robust DNA repair mechanisms including nucleotide and base excision repair systems, and elevated antioxidant enzyme expression that mitigates lethal damage and enables prolonged survival on dry surfaces [18,27]. When both Gram-positive and Gram-negative bacteria form biofilms on surfaces or medical devices, they show a considerable increase in their ability to handle stress. This is because extracellular polymeric substance (EPS) matrices physically protect embedded cells and scavenge reactive oxygen species, biofilm-specific upregulation of antioxidants and DNA repair genes through quorum sensing and stress regulons, and microenvironmental heterogeneity that shelters dormant subpopulations from exposure [20,21]. Fungal pathogens like *Candida albicans* and *Candida auris* exhibit complexity owing to their β-glucan-rich and glycoprotein-laden cell walls, mitochondrial compartmentalization that offers subcellular protection, and proficient DNA repair and photoreactivation mechanisms, particularly demonstrating significant resistance when embedded in biofilms [28,29,30]. *C. difficile* spores possess multilaminar protein coats, specialized chemically resistant peptidoglycan structures, and significant metabolic dormancy, resulting in an extraordinary resistance phenotype that allows prolonged survival on environmental surfaces [31,32].

**Table 1:** Risk-relevant healthcare pathogens.

Pathogen	Microbial Type	Cell Envelope Feature	Biofilm Capacity	DNA Repair Systems	Antioxidant Defenses	Environmental Persistence	Primary HAI Type (CDC/NHSN) *	Refs.
**Viruses**								
Influenza A virus	Enveloped viruses	Lipid envelope, no protective coat	No	None (RNA genome)	None (passive structure)	Moderate (hours to days)	RTI/Pneumonia (PNU1–3)	[16,17,33]
*SARS-CoV-2*	Enveloped viruses	Lipid envelope, no protective coat	Limited	None (RNA genome)	None (passive structure)	High (days to weeks)	RTI/Pneumonia (PNU1–3)	[23,24,34,35]
**Bacteria**								
*Staphylococcus aureus*	Gram-positive coccus	Thick peptidoglycan; no outer membrane	Strong	Constitutive (NER, BER, SOS)	High baseline (SOD, catalase, TRX)	High (weeks to months)	SSI/BSI/VAP	[18,25,27]
MRSA	Gram-positive coccus (MDR)	Thick peptidoglycan; no outer membrane	Strong	Constitutive (NER, BER, SOS)	High baseline (SOD, catalase, TRX)	Very high (5+ weeks)	SSI, BSI, VAP, SSTI	[13,16,27]
*Escherichia coli*	Gram-negative rod	LPS outer membrane; thin peptidoglycan	Moderate	Constitutive (NER, BER, SOS)	Moderate baseline	Moderate (hours to days)	UTI/CAUTI/BSI	[25,26,36]
*Pseudomonas aeruginosa*	Gram-negative rod	LPS outer membrane; thin peptidoglycan; mucoid capsule	Strong	Constitutive (NER, BER, SOS)	Moderate baseline	High (up to weeks)	VAP/BSI/SSI	[19,25,37]
*Clostridioides difficile* (spores)	Gram-positive bacillus	Multilaminar spore coat; dormant state	Biofilm-like aggregates	Inactive (dormant)	Inactive (dormant)	Very High (months)	GI infection (CDI)	[19,31,32]
**Fungi**								
*Candida auris*	Fungi	β-glucan/glycoprotein cell wall	Strong	Photoreactivation, excision repair	Enhanced baseline (SOD, catalase)	High (weeks to months)	BSI/CLABSI/ Surface colonization	[22,28,32]
*Candida albicans*	Fungi	β-glucan/glycoprotein cell wall	Very strong	Photoreactivation, excision repair	Enhanced baseline (SOD, catalase)	Moderate (hours to days)	BSI/CLABSI/UTI	[19,28,29,30]

* HAI type classifications follow the CDC/NHSN surveillance definitions for specific types of healthcare-associated infections [38,39]. Pathogen-HAI associations are based on NHSN surveillance data [3]. For far-UVC 222 nm resistance categories and quantitative fluence requirements for each pathogen across different environmental matrices, see Table 2.

### 3.2. Microbial Determinants of Susceptibility to Far-UVC 222 nm

#### 3.2.1. Viruses

**Figure 2:**
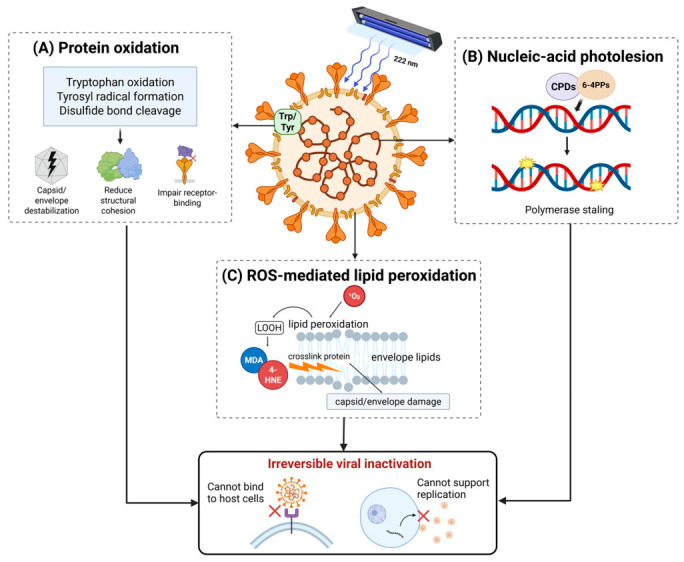
Far-UVC 222 nm inactivation mechanisms in enveloped viruses. Far-UVC 222 nm inactivates enveloped viruses through three convergent pathways. (**A**) Protein oxidation: 222 nm photons are preferentially absorbed by tryptophan (Trp) and tyrosine (Tyr) residues, inducing photooxidation, tyrosyl radical formation, and disulfide bond cleavage that destabilize the capsid/envelope and impair receptor binding. (**B**) Nucleic acid photolesions: CPDs and 6-4 PPs stall polymerase progression and arrest replication. Photoreactivation is suppressed at 222 nm, rendering lesions irreversible. (**C**) ROS-mediated lipid peroxidation: ^1^O_2_, hydroxyl radicals (·OH), and superoxide (O_2_^−^) from type II photosensitization peroxidize envelope lipids, generating lipid hydroperoxides (LOOH) and protein cross-links. These three convergent pathways result in irreversible loss of viral infectivity. Image created with BioRender.com. Prismasari, S. (2026) https://BioRender.com/anx2gds (accessed on 10 May 2026).

Far-UVC 222 nm inactivates viruses through photochemical and oxidative damage to nucleic acids, structural proteins, and lipid envelopes (Figure 2). Viruses lack metabolic and enzymatic repair systems, rendering photon-induced damage effectively irreversible [17,34]. Specifically, direct photon absorption by viral genomes produces cyclobutane pyrimidine dimers (CPDs) and pyrimidine–pyrimidone (6–4) photoproducts (6–4PPs), which disrupt base pairing and block polymerase progression [17]. However, nucleic acid damage alone does not fully account for virucidal efficacy at 222 nm compared to that at 254 nm [40,41].

Protein photochemistry is considered the principal mechanism of inactivation at 222 nm. Peptide bonds and aromatic residues including tryptophan and tyrosine exhibit absorption maxima near this wavelength [24,42]. Exposure to far-UVC 222 nm results is thought to result in various reactions including tryptophan oxidation to N-formylkynurenine, tyrosyl radical formation, and disulfide bond cleavage, which are proposed to destabilize the capsid architecture and impairment of the receptor-binding capacity for host cell entry [24,42]. These mechanisms have been characterized primarily through protein photochemistry studies; direct visualization of individual modifications in intact virions under 222 nm exposure remains limited. Additionally, functional assays have demonstrated dose-dependent reductions in receptor binding, consistent with the oxidative modification of aromatic residues and structural loss [41].

Additionally, photoexcited viral chromophores generate singlet oxygen (^1^O_2_) that initiates protein carbonylation, lipid peroxidation, and crosslinking [36]. Virions cannot reverse oxidative lesions, which result in a permanent loss of infectivity [35,36]. Notably, 222 nm light undergoes strong absorption in the stratum corneum and ocular tear film, limiting penetration to living cells and minimizing mutagenic DNA damage risk [17,43]. This safety profile supports continuous deployment in occupied healthcare environments, contrasting with conventional 254 nm UVC systems.

#### 3.2.2. Bacteria

**Figure 3:**
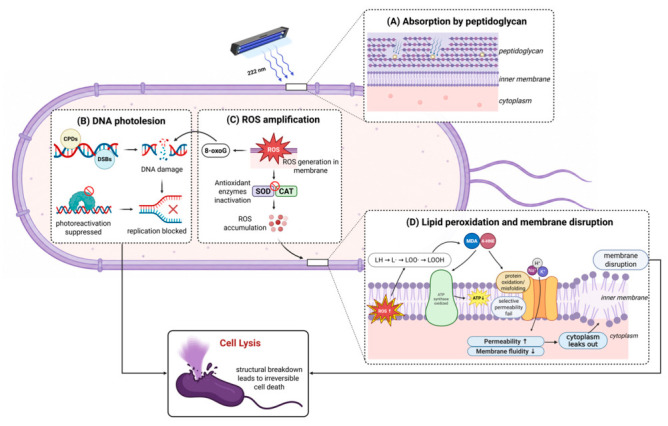
Far-UVC 222 nm inactivation mechanisms in bacteria (Gram-positive). Far-UVC 222 nm inactivates bacteria through interconnected mechanisms targeting DNA and membranes. (**A**) Peptidoglycan absorption: the cell wall partially attenuates 222 nm photons, with greater attenuation in Gram-positive (20–35 nm peptidoglycan) than Gram-negative bacteria (2–7 nm peptidoglycan with LPS outer membrane). (**B**) DNA photolesions: CPDs, DSBs, and 8-oxoG accumulate; photoreactivation is suppressed at 222 nm. (**C**) ROS generation and antioxidant ablation: ^1^O_2_, ·OH, and O_2_^−^ accumulate intracellularly; oxidative carbonylation of SOD and CAT ablates antioxidant defenses. (**D**) Membrane collapse: lipid peroxidation (LH → L· → LOO· → LOOH) generates malondialdehyde (MDA) and 4-hydroxynonenal (4-HNE); oxidative damage to porins and ATP synthase disrupts permeability and energy metabolism, leading to ATP depletion, ion imbalance, and bacterial lysis. Image created with BioRender.com. Prismasari, S. (2026) https://BioRender.com/anx2gds (accessed on 10 May 2026).

Bacterial inactivation by 222 nm light results from convergent action of direct photochemical DNA damage and reactive oxygen species (ROS)–mediated oxidative stress (Figure 3). Direct photon absorption by genomic DNA generates CPDs and double-stranded breaks (DSBs) that preferentially accumulate in AT-rich genomic regions [31]. CPDs block DNA polymerase processivity and halt replication fork progression [36], whereas DSBs cause irreparable genomic fragmentation. Notably, photolyases cannot repair CPDs under 222 nm light, unlike damage caused by conventional 254 nm UVC [44]. Similarly, ROS-mediated base oxidation, particularly 8-oxoguanine (8-oxoG) formation and thymine glycol adducts, disrupts DNA function and activates endonucleases [26,36].

Concurrent with DNA photodamage, 222 nm irradiation catalyzes rapid intracellular accumulation of superoxide (O_2_^−^), hydroxyl radicals (·OH), and singlet oxygen (^1^O_2_) [36]. Oxidative carbonylation of superoxide dismutase (SOD) and catalase (CAT), the primary bacterial ROS scavengers, directly ablates the antioxidant defense and establishes self-propagating oxidative cycles [36]. Notably, intracellular ROS levels in *S. aureus* (MRSA) are significantly higher following 222 nm irradiation than after equivalent germicidal doses of 254 nm UVC, and these elevated ROS persist even after photoreactivation, resulting in substantially lower proliferative recovery despite comparable levels of CPD repair between the two wavelengths [45]. This observation indicates that ROS-mediated oxidative injury, rather than unrepaired DNA photolesions alone, is a critical determinant of the superior bactericidal permanence of 222 nm far-UVC. Moreover, ROS-mediated damage simultaneously compromises DNA repair systems, rendering recovery ineffective [31,44].

Additionally, ROS-driven lipid peroxidation of membrane polyunsaturated fatty acids generates reactive carbonyl species that destabilize the lipid bilayer [26]. ·OH and ^1^O_2_ attack fatty acid chains and oxidize membrane proteins including porins and ATP synthase complexes, causing conformational collapse and loss of selective permeability [26,36]. Impaired oxidative phosphorylation depletes cellular ATP, preventing compensatory ion pumping, and precipitating an osmotic imbalance [31]. This progressive membrane deterioration is proposed to culminate in uncontrolled permeability, cytoplasmic leakage, and bacterial lysis [26,36]. It should be noted that the sequential presentation of these damage pathways (DNA lesions, ROS accumulation, and membrane failure) reflects a narrative convention; in practice, these processes likely operate simultaneously and synergistically, with the relative contribution of each pathway varying across bacterial species and environmental conditions.

The differential susceptibility of bacteria to 222 nm inactivation is closely linked to cell envelope composition. Among Gram-negative bacteria, species such as *E. coli* and *P. aeruginosa*, which possess lipopolysaccharide-rich outer membranes and thin peptidoglycan layers, undergo rapid ROS-mediated disintegration and endotoxin release upon far-UVC exposure [26]. By contrast, among Gram-positive species, organisms such as *S. aureus* and *E. faecium*, fortified with dense peptidoglycan layers and constitutively expressing DNA repair systems, including nucleotide and base excision repair, exhibit partial resistance [31]. Their thick peptidoglycan matrix slows ROS penetration and reduces the membrane damage rate, while higher baseline antioxidant defenses allow the partial attenuation of ROS before irreversible damage occurs [31]. Bacterial endospores represent an extreme case and display exceptional recalcitrance in response to far-UVC inactivation. Their multilaminar protective coats and profound metabolic dormancy prevent active repair processes, requiring substantially elevated fluences compared with vegetative cells [31].

The mechanistic evidence described above derives predominantly from studies of model organisms, particularly *E. coli* (Gram-negative) and *S. aureus* (Gram-positive). The extent to which these pathways generalize across all species within each category remains to be fully established. Species-specific differences in membrane lipid composition, DNA repair enzyme repertoire, antioxidant expression, and cell wall architecture may modulate the relative contribution of each inactivation pathway. Future studies employing a broader range of clinically relevant species under standardized far-UVC exposure conditions would strengthen the mechanistic framework presented here.

#### 3.2.3. Fungi (Candida Species)

The available evidence on far-UVC 222 nm antifungal efficacy in healthcare settings is primarily derived from studies of Candida species, including *C. albicans*, *C. auris*, and *C. parapsilosis.* Owing to the enormous diversity of fungal cell wall composition, pigmentation, morphology, and stress-response systems, the findings presented in this section may not generalize to filamentous fungi, dermatophytes, or other yeast genera, and should be interpreted with this limitation in mind.

**Figure 4:**
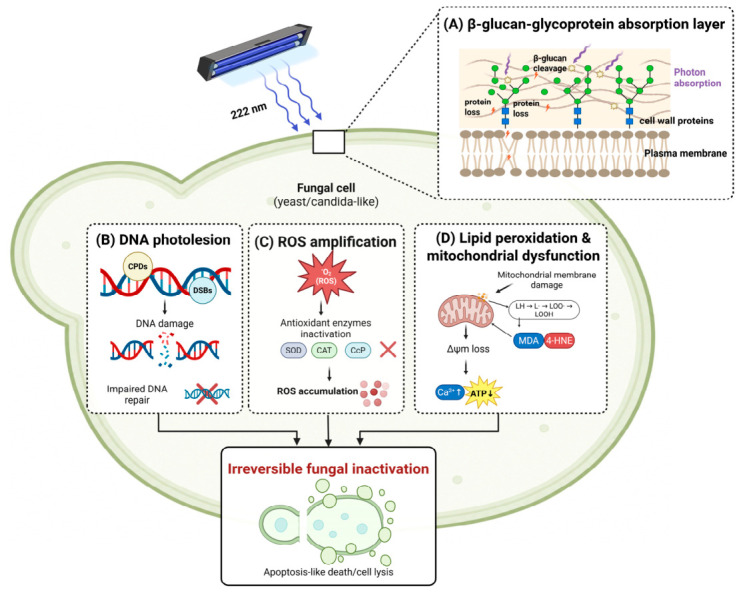
Far-UVC 222 nm inactivation mechanisms in fungi (Candida species). Far-UVC 222 nm inactivates fungi through interconnected photochemical and oxidative mechanisms. (**A**) Cell wall absorption: β-glucan-glycoprotein chromophores partially attenuate 222 nm photons, conferring greater resistance than bacterial envelopes. (**B**) DNA photolesions: CPDs and DSBs accumulate; photoreactivation is suppressed despite functional photolyase enzymes. (**C**) ROS amplification: ^1^O_2_ from type II photosensitization, with ·OH and O_2_^−^, overwhelms SOD and CAT through oxidative carbonylation. (**D**) Mitochondrial collapse: peroxidation of cardiolipin and phosphatidylethanolamine depolarizes mitochondrial membrane potential (Δψm), depletes ATP, and triggers intracellular Ca^2+^ accumulation and apoptotic-like programmed cell death. Evidence is primarily from *C. albicans, C. auris, and C. parapsilosis*; findings may not generalize to other fungal taxa. Image created with BioRender.com. Prismasari, S. (2026) https://BioRender.com/anx2gds (accessed on 10 May 2026).

As shown in Figure 4**,** far-UVC 222 nm radiation exhibits potent antifungal activity through coordinated photochemical and oxidative mechanisms targeting multiple cellular compartments [19]. In Candida species, absorption of 222 nm photons by fungal chromophores in glycoprotein-β-glucan complexes generates CPDs and DSBs in genomic DNA [19,29]. Dense cell wall polymers and intracellular heat shock proteins provide partial protection by limiting photon penetration, conferring greater resistance than bacterial cells; however, unrepaired DNA lesions accumulate beyond the photoreactivation and excision repair capacity of these fungal cells, resulting in genomic damage and replication cessation [19].

^1^O_2_ generation through type II photochemical reactions initiates lipid peroxidation and protein oxidative modification [19]. The oxidative carbonylation of critical antioxidant enzymes including superoxide dismutase and catalase, impairs the capacity of fungal cells to detoxify endogenously generated ROS, thus establishing self-perpetuating oxidative cycles that overwhelm antioxidant defenses [28].

Additionally, ROS-driven lipid peroxidation of mitochondrial membranes, particularly of cardiolipin and phosphatidylethanolamine, propagates free-radical chain reactions, generating lipid hydroperoxides and reactive aldehydes [19]. The accumulation of oxidation products compromises membrane structural integrity and selective permeability, impairing electron transport chain function, and disrupting oxidative phosphorylation. Subsequently, mitochondrial membrane potential (Δψm) progressively depolarizes, and aerobic ATP synthesis ceases, resulting in bioenergetic collapse and ATP depletion [19,29].

ATP depletion in turn prevents the completion of DNA repair and calcium extrusion, resulting in increased intracellular calcium levels that activate apoptotic cascades [29]. Thus, the convergence of unrepaired genomic damage, ROS-mediated enzyme inactivation, mitochondrial dysfunction, bioenergetic failure, and apoptotic activation ensures irreversible fungal inactivation [19,29].

### 3.3. Evidence Synthesis of Resistance Categories and Matrix Factors

Fluence requirements are directly correlated with microbial biology, as evidence synthesis shows that far-UVC resistance spans four orders of magnitude across pathogens. Based on fluence requirements for ≥99.9% (3-log) inactivation, pathogens were classified into four resistance categories as follows. These categories represent operational groupings intended to facilitate cross-pathogen comparison and should not be interpreted as absolute thresholds; susceptibility exists along a continuum, and individual species may fall near category boundaries depending on experimental conditions. The fluence values reported across studies reflect variable methodologies, and the apparent precision of the numerical ranges should be interpreted with caution given inter-study heterogeneity in inoculum preparation, surface material, and fluence measurement. Table 2 presents these resistance categories based on far-UVC 222 nm efficacy data across healthcare pathogens. Enveloped viruses (influenza A virus, SARS-CoV-2, coronaviruses) and airborne Gram-negative bacteria exhibit **Low resistance** at <3 mJ/cm^2^ owing to the absence of protective structures, low ROS-scavenging, and lack of enzymatic repair [16,33]. Specific examples include *P. aeruginosa* requiring approximately 1 mJ/cm^2^ to achieve 99% reduction, human coronavirus OC43 (HCoV-OC43) requiring 0.48–1.2 mJ/cm^2^ for 95–98% reduction, and influenza A virus (H1N1) requiring 1.6–2 mJ/cm^2^ for greater than 95% reduction [16,17,23,25].

**Moderate resistance** (3–10 mJ/cm^2^) characterizes planktonic Gram-positive bacteria on surfaces, reflecting peptidoglycan protection and inherent DNA repair mechanisms [18,27]. Biofilm-embedded pathogens and fungi exhibit **High resistance** (10–50 mJ/cm^2^), facilitated by EPS matrix protection and the upregulation of stress responses [19,25]. Spore-formers like *C. difficile* (>1000 mJ/cm^2^) and mature biofilms **Very High resistance** (>50 mJ/cm^2^). This exceptional resistance arises from their multilaminar coats and capacity for metabolic dormancy [31,32].

Far-UVC 222 nm efficacy varies markedly by environmental matrix. Airborne pathogens require low fluences (<3 mJ/cm^2^) with rapid kinetics and minimal biofilm interference, while surface-bound forms demand 3–100× higher doses owing to ROS-scavenging biofilms and surface shielding effects [20,46]. *E. coli* airborne inactivation occurs at 1.4–21 mJ/cm^2^ versus 12.5–19.48 mJ/cm^2^ on surfaces (10–14× increase), and *P. aeruginosa* shows a similar 30× escalation, reflecting surface attachment-triggered biofilm gene expression [19,21,25].

Real-world polymicrobial biofilms contrast sharply with laboratory monocultures. Continuous ward exposure (~513 mJ/cm^2^/day) yields 55–77% bioburden reduction, clinic surfaces (108 mJ/cm^2^) achieve 81.4%, and waiting rooms (~0.4 mJ/cm^2^/cycle) reach 70.5% over 14 weeks via cumulative dosing [20,46]. Far-UVC exposure of spittoon deposits at 3.6–13.5 mJ/cm^2^ achieved only 72% reduction, as organic shielding substantially attenuates UV penetration and ROS activity [47]. In a large occupied dental clinic containing 24 treatment chairs and equipped with 16 ceiling-mounted 222 nm fixtures, airborne culturable bacteria were reduced by 39.5% (95% CI: 19–60%), equivalent to 6.5 additional air changes per hour (eACH) above the existing 10 ACH mechanical ventilation [48]. Projections from this study indicate that in healthcare spaces with lower baseline ventilation (<3 ACH), the same installation would achieve greater than 70% airborne bacterial reduction, highlighting the inverse relationship between existing ventilation capacity and the marginal benefit provided by far-UVC air treatment. Community-level heterogeneity, not single-pathogen kinetics, determines field performance [20].

**Table 2:** Synthesized evidence of far-UVC 222 nm efficacy against healthcare-associated pathogens in airborne and surface environments.

Pathogen (Species)	Matrix	Fluence Range (mJ/cm^2^)	Efficacy (% Reduction)	Resistance Category	Protective Mechanism	References
Influenza A virus (H1N1)	Airborne	1.6–2	>95%	Low	Lipid envelope only; no enzymatic repair	[16,33]
SARS-CoV-2 (MS2 surrogate)	Airborne	0.068–2.5	99%	Low	Simple capsid; no enzymatic repair	[32,49]
	Surface	1.84	80%	Low	Simple capsid; no enzymatic repair	[49]
SARS-CoV-2	Surface	2.5–3	87–99.7%	Low	Lipid envelope; no enzymatic repair	[34,35]
HCoV-229E (α-coronavirus)	Airborne	1.7	99.9%	Low	Lipid envelope; no enzymatic repair	[17]
HCoV-OC43 (β-coronavirus)	Airborne	0.48–1.2	95–98%	Low	Lipid envelope; no enzymatic repair	[17,23]
*Staphylococcus aureus*	Airborne	1.68–23	95–98%	High	Thick peptidoglycan; Gram-positive stress response	[25,40]
	Surface	9.3–12.5	99.9%	Moderate	Thick peptidoglycan; surface-associated microcolonies	[19,50]
Methicillin-Resistant *Staphylococcus aureus* (MRSA)	Surface	2–20	>99.97%	High	Thick peptidoglycan; strong biofilm capacity; MDR-linked stress responses	[13,19,27,32,37]
*Escherichia coli*	Airborne	1.4–21	99%	High	LPS outer membrane; thin peptidoglycan; limited antioxidants	[51,52,53,54,55]
	Surface	12.5–19.48	99.9%	High	Early biofilm and microcolony formation; EPS shielding	[19,50,56]
*Staphylococcus epidermidis*	Airborne	0.437–47	39–99.9%	High	Gram-positive wall; variable stress-response activation	[51,53,57]
	Surface	9	32.43%	Moderate	Early biofilm formation; EPS begins to protect	[58,59]
*Staphylococcus hominis*	Airborne	9.7	99.9%	Moderate	Gram-positive wall; similar stress responses to coagulase-negative staphylococci	[58]
*Pseudomonas aeruginosa*	Airborne	~1	99%	Low	LPS outer membrane; thin peptidoglycan; limited antioxidants	[25]
	Surface	18.6–33.97	76–99%	High	Strong biofilm; mucoid capsule; EPS matrix	[19,37]
*Clostridium difficile*	Surface	>1000	99.9%	Very high	Multilaminar spore coats; dormant core	[19,32]
Vancomycin-resistant *Enterococcus* (VRE)	Surface	15–20	99.9%	High	Thick peptidoglycan; MDR-associated stress responses	[32]
*Candida albicans*	Surface	8.6–50	99.9%	Very high	β-glucan/glycoprotein cell wall; eukaryotic DNA repair; mitochondrial compartmentalization	[19,30,50]
*Candida auris*	Surface	15–20	99.9%	High	β-glucan wall; strong biofilm formation; enhanced antioxidants	[32]
Mixed surface pathogens	Surface (ward, mobile workstations)	~513 (per day)	55–77%	NA	Polymicrobial biofilms; organic soil; variable shading	[20]
	Surface (plastic chairs, waiting room)	~0.4	70.5%	NA	Thin biofilms and environmental deposits; repeated low-dose exposure	[20]
	Surface (clinic surface)	108	81.4%	NA	Polymicrobial communities; mixed biofilm maturity	[46]
	Dental spittoon	3.6–13.5	72%	NA	Dried respiratory deposits; biofilm-like aggregates; high organic load	[47]
Mixed airborne bacteria	Airborne (dental clinic)	~6.5	39.5%	NA	Polymicrobial airborne; 16 ceiling-mounted fixtures; 10 ACH baseline	[48]

NA, not applicable. Resistance categories are defined by far-UVC fluence required for approximately 99.9% (3-log) inactivation as follows: **Low** (<3 mJ/cm^2^), **Moderate** (3–10 mJ/cm^2^), **High** (10–50 mJ/cm^2^), and **Very High** (>50 mJ/cm^2^). Efficacy values reflect the endpoints reported in the cited primary studies; not all studies achieved or reported 3-log inactivation. Variation in inoculum preparation, surface material, organic load, and fluence measurement methods limit direct cross-study comparison.

## 4. Discussion

The evidence reviewed facilitates a microbiology-informed understanding of far-UVC disinfection performance. Differential susceptibility closely follows bacterial taxonomy and cell envelope architecture, with Gram-negative bacteria generally more susceptible than Gram-positive organisms and spore formers representing an extreme of resistance. Gram-negative bacteria possess LPS outer membranes and thin peptidoglycan layers that are vulnerable to oxidative damage, while Gram-positive bacteria benefit from thick peptidoglycan barriers, constitutive DNA repair systems, and robust antioxidant defenses that delay lethal injury [18,25,26]. These patterns are consistent with long-term evolutionary exposure to environmental UV stress, particularly in soil-dwelling and spore-forming organisms that have retained highly protective structures [16,31].

The differential mechanisms of pathogen inactivation at 222 nm compared with conventional germicidal 254 nm UVC merit explicit consideration, as these differences have direct implications for disinfection strategy. At 254 nm, the primary target is nucleic acid; DNA absorbs strongly at this wavelength, producing CPDs and 6-4PPs that can be partially reversed by photoreactivation repair in organisms possessing photolyase enzymes [36,44]. By contrast, at 222 nm, peptide bonds and aromatic amino acid residues (particularly tryptophan and tyrosine) exhibit substantially stronger absorption, making protein damage the predominant inactivation mechanism [24,42]. This wavelength-dependent shift in primary target has two important consequences. First, protein-mediated damage, including capsid destabilization, receptor-binding impairment, and enzyme inactivation, is effectively irreversible because cells and virions lack dedicated protein repair pathways analogous to DNA repair systems. Second, 222 nm irradiation generates higher levels of reactive oxygen species through type II photosensitization reactions, amplifying oxidative damage to membranes and intracellular components [36]. Critically, ROS generated at 222 nm have been shown to suppress photoreactivation in *E. coli* [36,44] and in *S. aureus* (MRSA), where proliferative recovery following photoreactivation is significantly lower after 222 nm irradiation than after 254 nm exposure, despite comparable CPD formation and equivalent photolyase-mediated repair between the two wavelengths [45].

Because the DNA lesion burden and its repair are equivalent, the reduced recovery at 222 nm can be attributed to persistent ROS-mediated oxidative damage to proteins, lipids, and membrane components that cells cannot reverse through photoreactivation. This convergence of irreversible protein damage, enhanced ROS generation, and photoreactivation suppression provides the mechanistic basis for the potent germicidal activity of 222 nm light and explains why pathogens equipped with photoreactivation capacity do not benefit from this repair mechanism under far-UVC exposure.

A significant finding is the robust correlation among multidrug resistance, biofilm development, and decreased susceptibility to far-UVC. Numerous high-priority MDR bacteria, such as MRSA, *P. aeruginosa*, *A. baumannii*, VRE, and *C. auris*, exhibit robust biofilm formation and share similar stress-response networks that provide resistance to both antibiotics and UV-induced harm [20,21]. However, multidrug resistance itself does not appear to confer cross-protection against far-UVC 222 nm irradiation. Clinical MDR isolates from multiple bacterial species exhibited 222 nm inactivation kinetics comparable to those of their antibiotic-susceptible reference counterparts under identical fluence conditions. A notable exception was a pan-resistant *Klebsiella pneumoniae* isolate, in which residual tolerance may be attributable to capsular polysaccharide-mediated UV shielding rather than to resistance mechanisms shared with antibiotic efflux or enzymatic inactivation pathways [60]. This distinction is clinically important, as it suggests that far-UVC 222 nm may retain efficacy against MDR pathogens that are increasingly refractory to conventional chemical antimicrobials. The upregulation of antioxidant enzymes, DNA repair mechanisms, and efflux systems in biofilms provide cross-protection against ROS and genotoxic chemicals, whereas EPS matrices and microenvironmental heterogeneity protect subpopulations from exposure [18,28]. Infection control programs suggest that clinical settings burdened with a significant presence of MDR biofilm-forming bacteria may necessitate elevated cumulative far-UVC doses, strategic fixture placement, and the incorporation of manual cleaning and chemical disinfection to attain the necessary risk mitigation.

Furthermore, real-world healthcare biofilms are complex, polymicrobial communities in which interspecies interactions substantially influence collective behavior and stress tolerance. Within these communities, microorganisms communicate through quorum-sensing signaling molecules, engage in metabolic cooperation, and establish cooperative resistance mechanisms that may further modulate susceptibility to far-UVC beyond what single-species laboratory studies predict [21]. For example, EPS produced by one species may shield neighboring species from UV penetration, and metabolically dormant subpopulations within mixed biofilms may serve as reservoirs for regrowth following sublethal exposure. Existing characterizations of microbial populations on healthcare surfaces provide valuable context for understanding the substantial gap between laboratory-derived inactivation kinetics and real-world field performance, where 55–81% bioburden reductions are typical despite cumulative doses that would achieve near-complete inactivation in monoculture experiments [20,46].

The exceptional resilience of *C. difficile* spores underscores a significant limitation for far-UVC implementation. Laboratory research suggests that far-UVC doses surpassing 1000 mJ/cm^2^ may be necessary for effective spore inactivation on surfaces, which conflicts with existing exposure limits for occupied environments and standard operational parameters [31,32]. This finding emphasizes current recommendations that sporicidal chemical agents and mechanical cleaning are crucial for *C. difficile* management, while far-UVC should be considered an adjunctive measure to diminish vegetative bacterial and fungal loads and disrupt the transmission of non-spore-forming organisms, rather than a singular solution for all pathogen categories.

Practical considerations for clinical deployment of far-UVC 222 nm should account for the pathogen-specific resistance hierarchy established by this review. For airborne transmission control, ceiling-mounted continuous far-UVC systems appear well suited for occupied patient care areas, operating rooms, and waiting rooms, where low cumulative fluences can substantially reduce viral and susceptible bacterial aerosol loads [20,43,48]. Real-world validation of this approach in an occupied 24-chair dental clinic demonstrated that 16 ceiling-mounted 222 nm fixtures, operating well below ACGIH threshold limit values (average occupant dose 25.35 mJ/cm^2^ over 8 h), achieved a 39.5% reduction in airborne culturable bacteria equivalent to 6.5 additional air changes per hour, with no adverse occupant feedback over two years of continuous operation [48]. The finding that efficacy increases substantially at lower baseline ventilation rates (projected >70% reduction at <3 ACH) suggests that far-UVC 222 nm may provide the greatest marginal benefit in older or resource-limited healthcare facilities where HVAC upgrades are impractical. For surface decontamination, handheld or portable far-UVC devices may complement terminal cleaning protocols by providing targeted treatment of high-touch surfaces, although the higher fluences required for biofilm-embedded organisms and Gram-positive bacteria may necessitate extended exposure times or repeated treatment cycles [46]. Combination strategies integrating far-UVC with manual cleaning (to remove organic soil that attenuates UV penetration), chemical disinfection (to address spore-forming organisms), and conventional 254 nm UVC (for unoccupied terminal disinfection) are likely to offer the broadest pathogen coverage [7,8]. However, evidence supporting specific dose protocols and optimal device configurations for different clinical settings remains limited, and facility-specific validation studies are warranted before widespread implementation. Monitoring programs that track changes in microbial susceptibility over time should also be considered to detect any emergence of UV-adapted populations.

Whereas the resistance categories described above reflect intrinsic structural and biochemical properties, the potential for acquired resistance through adaptive evolution under repeated sublethal exposure represents a distinct and currently underexplored concern. Experimental evolution studies in *E. coli* show that changes to DNA repair pathways, transcriptional machinery, and membrane components can render the organism more resistant to UV light in just a few generations [36,44]. In clinical settings, high rates of bacterial mutation, repeated cycles of sublethal exposure, and the ability to respond to stress in a way that makes bacteria more resistant may help bacteria adapt similarly. This is particularly applicable to Gram-negative bacteria presently classified as low resistance [25]. This scenario highlights the imperative for surveillance initiatives that track temporal fluctuations in susceptibility, encompassing molecular monitoring of UV-resistance-associated mutations, and promote comprehensive disinfection strategies that combine far-UVC 222 nm with chemical and physical methods to reduce the risk of directional selection.

This review has several limitations. The synthesis relies on published experimental and field studies, which may overrepresent positive findings or well-performing devices. Direct head-to-head comparisons of MDR and susceptible strains are limited, and data on mature, clinically derived biofilms remain sparse. Heterogeneity in experimental conditions, fluence measurement, and reporting complicates quantitative comparison across studies. In addition, most field evaluations report aggregate microbial outcomes rather than organism-specific data, making it difficult to link efficacy directly to pathogen biology in real-world settings [20,46]. Several real-world factors may further attenuate far-UVC efficacy compared with controlled laboratory conditions, including geometric shadowing by furniture, equipment, and patient positioning that prevents direct UV exposure of contaminated surfaces; organic soil and proteinaceous material that absorb UV photons and reduce effective surface doses; lamp output variability and gradual degradation over operational lifetime that affect delivered fluence; distance-dependent dose attenuation following the inverse-square law; and environmental parameters such as temperature and relative humidity that may modulate inactivation kinetics [20,46,47]. Future research should prioritize standardized fluence and outcome reporting, genomic and transcriptomic studies of resistance evolution under far-UVC 222 nm exposure, and carefully controlled investigations of MDR biofilms and polymicrobial communities on clinically relevant surfaces.

## 5. Conclusions

Healthcare settings can enhance disinfection through the use of far-UVC 222 nm to continuously reduce airborne viruses and susceptible vegetative bacteria. Microbiological studies indicate that the efficacy of pathogen disinfection is significantly affected by taxonomic classification, cellular envelope composition, biofilm condition, and specific survival strategies such as spore formation. Enveloped respiratory viruses and airborne Gram-negative bacteria can be inactivated at low fluences appropriate for occupied environments; however, Gram-positive bacteria, fungal pathogens, biofilm-embedded cells, and particularly bacterial spores necessitate higher doses or combined interventions.

Comprehending pathogen-specific resistance patterns and local epidemiology will facilitate the safe deployment of far-UVC. When utilized alongside manual cleaning and chemical disinfection, surface-directed or portable devices can enhance the decontamination of selected high-touch areas, whereas continuous upper-room or ceiling-mounted systems can substantially reduce the risk of airborne transmission. Nonetheless, far-UVC 222 nm alone is unlikely to manage highly resistant entities such as *C. difficile* spores and mature, organic-laden biofilms, necessitating sporicidal agents and thorough mechanical cleaning.

A microbiology-informed far-UVC disinfection approach acknowledges these limitations and employs the technology in its most effective applications. Healthcare facilities can systematically and empirically enhance HAI environmental control by incorporating far-UVC 222 nm with traditional infection prevention strategies, tailoring implementation to local pathogen profiles, and monitoring for changes in susceptibility.

## Data Availability

No new data was generated as part of this work.

## Abbreviations

The following abbreviations are used in this manuscript:

4-HNE	4-hydroxynonenal
6-4PPs	Pyrimidine–pyrimidone (6-4) photoproducts
8-oxoG	8-oxoguanine
ACH	Air changes per hour
eACH	Equivalent air changes per hour
ACGIH	American Conference of Governmental Industrial Hygienists
ATP	Adenosine triphosphate
BER	Base excision repair
BSI	Bloodstream infection
Ca^2+^	Calcium ion
CAT	Catalase
CAUTI	Catheter-associated urinary tract infection
CDC	Centers for Disease Control and Prevention
CDI	*Clostridioides difficile* infection
CI	Confidence interval
CLABSI	Central line-associated bloodstream infection
CPDs	Cyclobutane pyrimidine dimers
DNA	Deoxyribonucleic acid
DSBs	Double-strand breaks
EPS	Extracellular polymeric substance
Far-UVC	Far-ultraviolet C
GI	Gastrointestinal
HAIs	Healthcare-associated infections
HVAC	Heating, ventilation, and air conditioning
HCoV	Human coronavirus
LOOH	Lipid hydroperoxides
LPS	Lipopolysaccharide
MDA	Malondialdehyde
MDR	Multidrug-resistant
MRSA	Methicillin-resistant *Staphylococcus aureus*
NER	Nucleotide excision repair
NHSN	National Healthcare Safety Network
^1^O_2_	Singlet oxygen
·OH	Hydroxyl radical
O_2_^−^	Superoxide
PE	Phosphatidylethanolamine
PNU	Pneumonia (CDC/NHSN classification)
RNA	Ribonucleic acid
ROS	Reactive oxygen species
RTI	Respiratory tract infection
SARS-CoV-2	Severe acute respiratory syndrome coronavirus 2
SOD	Superoxide dismutase
SOS	Stress response regulator
SSI	Surgical site infection
SSTI	Skin and soft tissue infection
TRX	Thioredoxin
Trp	Tryptophan
Tyr	Tyrosine
UTI	Urinary tract infection
UV	Ultraviolet
UVC	Ultraviolet C
VAP	Ventilator-associated pneumonia
VRE	Vancomycin-resistant enterococci
Δψm	Mitochondrial membrane potential
